# Startle Auditory Stimuli Enhance the Performance of Fast Dynamic Contractions

**DOI:** 10.1371/journal.pone.0087805

**Published:** 2014-01-28

**Authors:** Miguel Fernandez-Del-Olmo, Dan Río-Rodríguez, Eliseo Iglesias-Soler, Rafael M. Acero

**Affiliations:** Learning and Human Movement Control Group, INEF Galicia, University of A Coruña, A Coruña, Spain; University of Salamanca- Institute for Neuroscience of Castille and Leon and Medical School, Spain

## Abstract

Fast reaction times and the ability to develop a high rate of force development (RFD) are crucial for sports performance. However, little is known regarding the relationship between these parameters. The aim of this study was to investigate the effects of auditory stimuli of different intensities on the performance of a concentric bench-press exercise. Concentric bench-presses were performed by thirteen trained subjects in response to three different conditions: a visual stimulus (VS); a visual stimulus accompanied by a non-startle auditory stimulus (AS); and a visual stimulus accompanied by a startle auditory stimulus (SS). Peak RFD, peak velocity, onset movement, movement duration and electromyography from pectoralis and tricep muscles were recorded. The SS condition induced an increase in the RFD and peak velocity and a reduction in the movement onset and duration, in comparison with the VS and AS condition. The onset activation of the pectoralis and tricep muscles was shorter for the SS than for the VS and AS conditions. These findings point out to specific enhancement effects of loud auditory stimulation on the rate of force development. This is of relevance since startle stimuli could be used to explore neural adaptations to resistance training.

## Introduction

Many sports require the ability to quickly react and contract muscles in response to a stimulus. For example, sports such as karate, boxing, or fencing require a rapid, and goal-directed movement as a reaction to the adversary movement, with muscle contraction times below 250 ms [1]–[3]. Under these situations, a high rate of force development (RFD) is crucial in order to achieve high levels of force and speed during the movement, since the maximal RFD determines the force that can be generated in the early phase of muscle contraction [4].

Fast reaction times and RFD are crucial for sport performance, however, little is known regarding the relationship between these parameters. Studies using reaction time paradigms, where subjects are asked to respond to an imperative stimulus, have reported a positive relationship between intensity of the imperative stimulus (“go” cue) and the force achieved during the motor response [5]–[7]. In these experiments, shorter reaction times were followed by a stronger motor response. Importantly, the subjects were asked to perform as quickly as possible but were not given explicit instructions to use maximal force in each response, which could explain the positive relationship between the parameters. In contrast, a recent study, using maximal isometric grip action in response to a “go” visual signal, showed no correlation between reaction times and RFD values [8]. Furthermore, previous studies have recorded motor responses that involve relatively small muscles (i.e. flexion-extension of index finger) and isometric contractions, but not multi-joint exercises, such as a *bench press*, that involve larger muscles and is performed in an explosive dynamic fashion.

Another important issue that has to be considered is that the nature of the stimulus that induces the motor response could affect the RFD during fast and forceful dynamic contractions. Several studies have suggested that auditory stimuli can enhance motor performance, for example, rhythmic auditory cues are able to induce the H-reflex during vertical jumps, allowing the subject to achieve higher vertical acceleration [9], while loud sounds delivered seconds before the contraction can increase the maximal pull of forearm flexors [10]. The most noticeable interaction between the auditory and motor systems is the startle reaction, where faster reaction times are observed when a startling acoustic stimulus (120 dB) is presented simultaneously with an imperative “go” stimulus [11]. The short reaction time (70 ms) and the unaffected electromyography pattern of the responses suggest that “the whole motor programme can be triggered [by the startle] without the expected command from the cerebral cortex” (Valls-Solé et al. 1999, p. 937). RFD has also been shown to be increased during a maximal isometric grip action when the imperative visual cues were accompanied by a startle auditory stimulus [8]. This could suggest that the ability to achieve the maximal RFD during a voluntary contraction could be affected by the intensity and modality of the imperative cue.

The aim of this study was to investigate the effects of auditory stimuli of different intensities on the rate of force development and reaction time, during the performance of a concentric bench-press exercise in subjects with resistance training experience, using kinematic and electromyography parameters. Concentric bench-presses were performed by trained subjects in response to three different conditions: a visual stimulus; a visual stimulus accompanied by a non-startle auditory stimulus; and a visual stimulus accompanied by a startle auditory stimulus. We hypothesized that a startle auditory stimulus will induce higher RFD and faster reaction times compared with visual or non-startle auditory stimuli. The validation of this hypothesis would be of relevance since startle stimuli could be used as a novel procedure to explore neural adaptations to resistance training.

## Materials and Methods

### Subjects

Thirteen young males participated in this study (age 25±3 years, height 1.81±6.87 m, weight 82.6±10.95 kg). The subjects were recruited from the Institute of Physical Education and Sport of A Coruña, Spain. All the subjects were healthy, physically active and have been implementing the *bench press* as an exercise into their training routine for at least the last three years. All subjects provided their written informed consent to participate in this study after being informed of the possible risks of the study. The experimental procedures conformed with the Declaration of Helsinki and were approved by the local ethics committee (Universidade de A Coruña).

### Procedure

**Figure 1:**
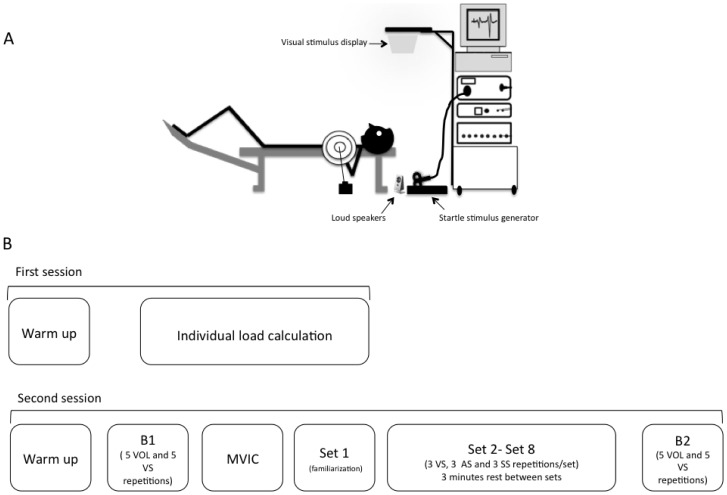
Experimental set-up and sessions. (A) Schematic representation of the experimental set-up. Visual stimuli were displayed in front of the subject and auditory and startle stimuli behind the subject's head. (B) Experimental sessions. MVIC  =  Maximal Voluntary Isometric Contraction; B1  =  Baseline block 1; B2  =  Baseline block 2.

Each subject participated in two experimental sessions separated by 1 week. The protocol and the set-up of the experiment are described schematically in Fig. 1.

The first session was conducted in order to determine the individual load for each subject and to achieve the highest mean propulsive power (MPP) during the concentric bench press exercise. When a light or medium load is lifted, a final phase is observed during which athletes apply force in the opposite direction to the load's motion. Thus, acceleration during this final period is lower than the effect of gravity. Therefore, the concentric phase of the movement can be divided into two phases: a propulsive (acceleration (a) higher than a >−9.81 m·s^−2^) and a braking phase (i.e. a<−9.81 m·s^−2^). Evaluating the propulsive phase avoids the underestimation of an individual's true neuromuscular potential when lifting light and medium loads [12].

The execution of a concentric bench press has been previously described [12]. Briefly, each subject was instructed to lower the bar to the chest and wait there until hearing the “go” signal from the evaluator. To avoid a rebound effect an interval of 1.5 seconds was used between the position of the bar over the chest and the “go” signal. The subjects were encouraged to lift the load as fast as they can for each repetition. Subjects were not allowed to bounce the bar off their chests or raise the shoulders or trunk off the bench.

The session started with a standardized warm-up that consisted of 5 minutes tempo run at low intensity, 5 minutes of joint mobility and one set of 10 repetitions of a concentric bench press with a 20 kg load. Following the warm-up, the subjects performed several sets of concentric bench presses of 4 repetitions each, with a rest period of 3 minutes. The initial load was set at 15 kg for all the subjects, and was progressively increased in 10 kg increments until the MPP was lower than the previous set. Thereafter, load was adjusted for the following sets by decrements of 5 kg and increments of 1 kg until each individual achieved their highest MPP.

The second session was conducted in order to determine the dynamic, kinematic and EMG parameters of the bench press performed under different conditions. The session started with the standardized protocol used in the first session following the first baseline block (B1). During this block the subjects were asked to perform 10 concentric bench press repetitions (the load was determined in the first experimental session). The initiation of the movement was randomly determined to be either voluntary (VOL condition) or in response to a visual imperative stimulus (VS condition). In the VOL condition the subjects initiated the movement voluntarily without any external imperative signal. The verbal instructions were “initiate the movement when you are ready”. The subjects were asked to initiate the movement within a maximum of 4 seconds after the verbal instructions. In the VS condition the subjects initiated the movement in response to a visual imperative stimulus - a white 25 cm2 square displayed on a black computer screen situated vertically in front of the subject's head (Fig. 1A). A warning auditory cue preceded the visual stimulus with a variable period of 2–4 s.

Three minutes later a five seconds maximal voluntary isometric contraction (MVIC) was performed by the subjects by pushing the bar away from their chest, during which the maximal electromyography activation of the pectoralis major and triceps muscles was recorded. Five minutes after the MVIC the subjects performed 8 sets of concentric bench presses. In all the sets and repetitions subjects were asked to initiate the movement in response to the visual imperative stimulus. In some trials the onset of the visual stimulus was simultaneous with an auditory-non startle stimulus consisting of a 750 Hz tone burst lasting 30 ms with intensity of 70 dB, while in the other trials the onset of the visual stimulus was simultaneous with a startling auditory stimulus with an intensity of 130 dB. We measured the stimulus intensity of each of the auditory stimuli using a type 2204 Bruel and Kjaer Impulse Precision Sound Level Meter. The subjects were instructed to concentrate on responding to the visual stimulus, regardless of the presence of the auditory stimuli. Each set consisted of 9 repetitions, 3 with only the visual stimulus (VS condition), 3 with the visual and auditory-non startle stimuli (AS condition) and 3 with the visual and startle stimuli (SS condition). The order of the trials was randomized with 3 minutes rest between sets. The first set was used to familiarize the subjects with the procedure and the loud sound and thus, excluded from the analysis.

In order to test whether the seven sets resulted in fatigue, the subjects performed a second baseline block (B2) identical to the first block, 5 minutes after the 7^th^ set.

### Kinematic recording

A dynamic measurement system (T-Force System, Ergotech, Murcia, Spain), consisting of cable-extension linear velocity transducers, was used. The cable was positioned in one extreme of the barbell. The T-Force System was connected to a computer using a 14-bit resolution analog-to-digital data acquisition board and integrated with a CED Power1401 amplifier (CED Power1401, Cambridge Electronic Design, Cambridge, UK) to record the barbell displacement in synchronization with the EMG recordings. The frequency sample was 1000 Hz.

The visual stimuli were displayed using Superlab software (Cedrus Corporation, CA, USA). Both auditory stimuli were delivered though a digital pulse (5 v) generated by the CED amplifier and connected to two loud speakers and a magnetic stimulation device. The auditory-non startle stimulus was emitted by the two loud speakers positioned 30 cm behind the subject's head. The startle auditory stimulus was produced by discharging the magnetic stimulus over a metallic platform positioned at a distance of 1 meter behind the subject's head (Fig. 1A), as previously described [12]. In order to synchronize the visual and auditory stimuli, the Superlab software was connected to the CED amplifier using a PCI card.

### Electromyography Recording

Electromyography (EMG) activity from the pectoralis major, triceps brachii and sternocleidomastoid (SCM) were recorded during the bench press exercise using Ag-AgCl bipolar surface electrodes. After skin preparation (shaved, abrasion, and cleaning with alcohol), the electrodes were coated with electrolytic gel and fixed over the middle of the muscle belly along the longitudinal axis, and the reference electrode was secured over the acromion of the right scapula. To reduce movement artefacts, the electrodes were taped firmly in place and a bandage was applied to the thigh to avoid cable movements. The electrodes were kept in place throughout the experimental session. The raw signal was amplified and filtered with a band-pass filter of 30–1 kHz (Digitimer, Welwyn Garden City, UK). Signals were digitized at 1000 Hz (CED Power1401, Cambridge Electronic Design, Cambridge, UK) and stored on a laboratory computer for off-line analysis.

### Data analysis

The following mechanic variables were recorded using specialized software (T-Force System, Ergotech, Murcia, Spain): Peak velocity (Vmax), peak of rate force development (RFDmax), barbell displacement and movement duration. The T-Force System, automatically calculated the relevant kinematic and kinetic parameters of every repetition, provided real time information on screen and stored data on disk for subsequent analysis. Vertical instantaneous velocity (*v*) was sampled at a frequency of 1000 Hz. The derived mechanical variables were calculated by the software as follows: displacement was obtained by integration of *v* data with respect to time; instantaneous RFD output resulted from the differentiation of the vertical applied force with respect to time.

In addition, the onset movement was defined as the time between the visual stimulus and the onset of the barbell movement.

The EMG parameters obtained were the Root Mean Square (RMS), onset latency and burst duration of pectoralis major and triceps brachii, and the time between the pectoralis and triceps burst (pect-tric). The RMS was normalized to the RMS obtained during the MVIC. Onset latency was defined as the time between the visual stimulus and the onset of the EMG activation. The onset and offset of EMG activity were visually determined by an interactive cursor of 1 ms resolution in combination with a rising threshold method using Signal software (Cambridge Electronic Design ltd.).

The movement delay (MD) was defined as the time between the onset of pectoralis EMG and the onset of movement.

### Statistical analysis

The analysis of the three conditions and seven sets was performed using the average values of all the trials. ANOVAs of repeated measured were performed with condition (VS, AS, SS) as a main factor for the following variables: Vmax; RFDmax; onset and duration of movement; barbell displacement; onset latency, duration and RMS of pectoralis and triceps activations. Post-hoc t tests were computed using Sidak correction for multiple comparisons. Wilcoxon test was used to analyze MD and time between pectoralis and triceps activations, since these variables violated the normality assumption necessary to conduct parametric statistical tests.

Pearson's correlation coefficients were calculated for each experimental condition to determine the relationship between onset EMG latency activation and the onset of maximal speed and RFD movement.

In order to explore the performance differences between voluntary and visual initiation of movement was and to determine whether seven sets led to fatigue effects, two-way ANOVAs of repeated measured were performed with block (Baseline 1 vs Baseline 2) and condition (VOL vs VS) as factors. The ANOVAs were performed for the variables Vmax and RFDmax. Post-hoc t tests were computed using Sidak correction for multiple comparisons.

All statistical analyses were performed using SPSS (SPSS, Chicago, IL). A P value≤0.05 was considered statistically significant.

## Results

### Startle effect on movement kinematics

Table 1 shows the mean values for the kinematic and EMG parameters measured for bench presses in each of the three conditions, VS, AS and SS.

**Table 1:** Kinematic and EMG meaurements.

	VS	AS	SS
**Kinematic measurements**			
Peak RFD (N/s)	1981.31±667	1953.22±547	2353.13±675
Peak Velocity (m/s)	1.73±0.24	1.73±0.24	1.76±0.23
Onset movement (ms)	365.46±45	289.29±45	241.06±43
Movement duration (ms)	491.56±72	509.08±85	463.93±70
Barbell displacement (cm)	53.55±6.78	52.93±6.44	53.71±6.53
**EMG**			
Onset sternocleidomastoid (ms)	–	–	71±24
Onset pectoralis (ms)	288.56±36	215.58±44	152.53±43
Duration pectoralis (ms)	450.68±98	427.47±85	414.07±90
Onset triceps (ms)	315.48±45	236.59±50	169.23±51
Duration triceps (ms)	416.52±89	401.31±76	387.84±81
Pect-triceps time (ms)	30.99±19	32.45±22	31.68±16
Movement delay (ms)	49.97±29	52.69±27	71.82±30
RMS pectoralis (% MVC)	59.18±20	59.89±19	60.45±20
RMS triceps (% MVC)	41.50±11	41.41±9	42.01±10

Mean values for the kinematic and EMG parameters measured for bench presses in each of the three conditions, VS, AS and SS.

**Figure 2:**
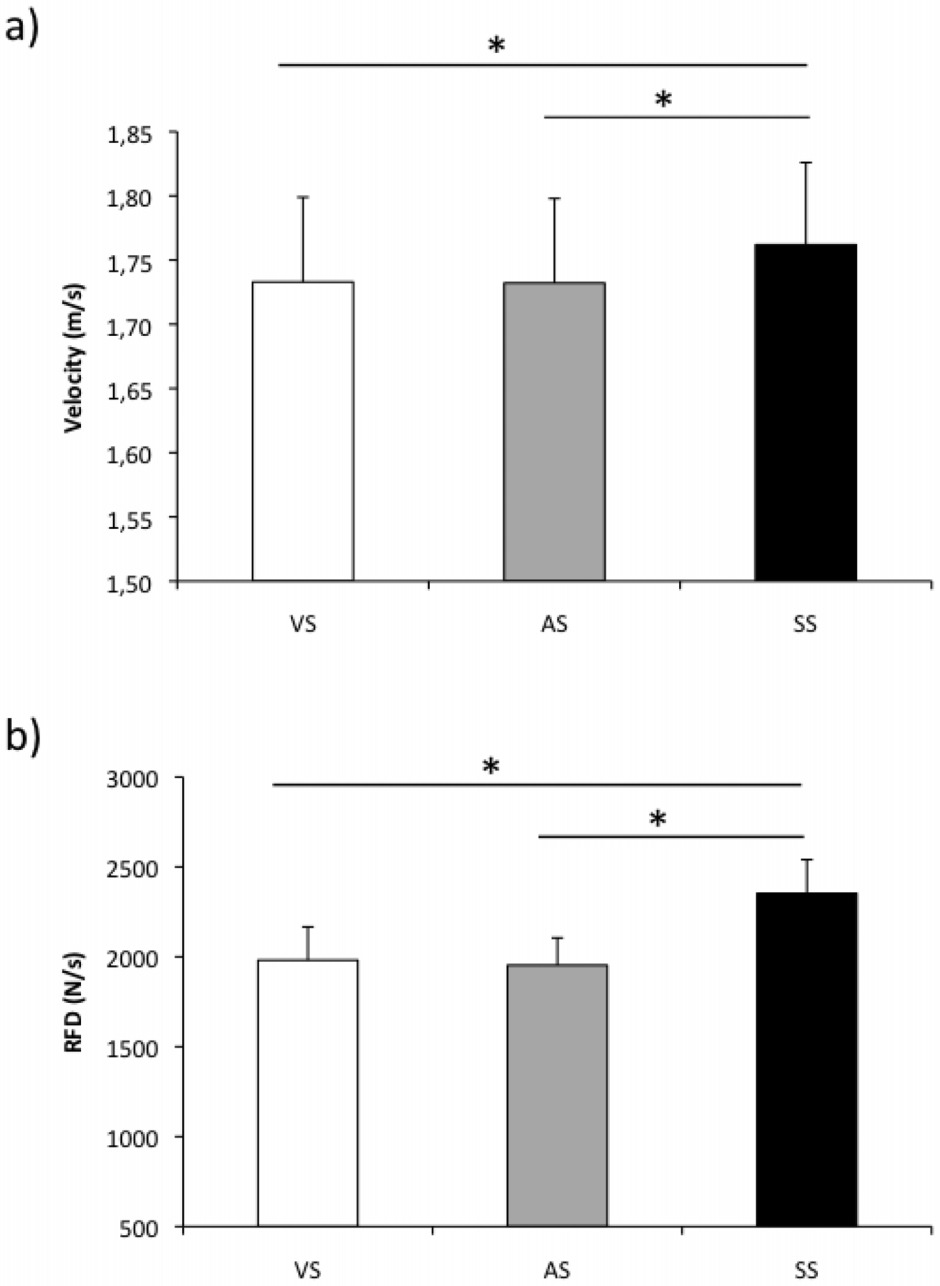
Kinematic values during bench press. Mean values of Vmax (a) and RFDmax (b) for conditions (VS) imperative visual stimulus; (AS) visual plus auditory stimulus; (SS) visual plus startle auditory stimulus.

**Figure 3:**
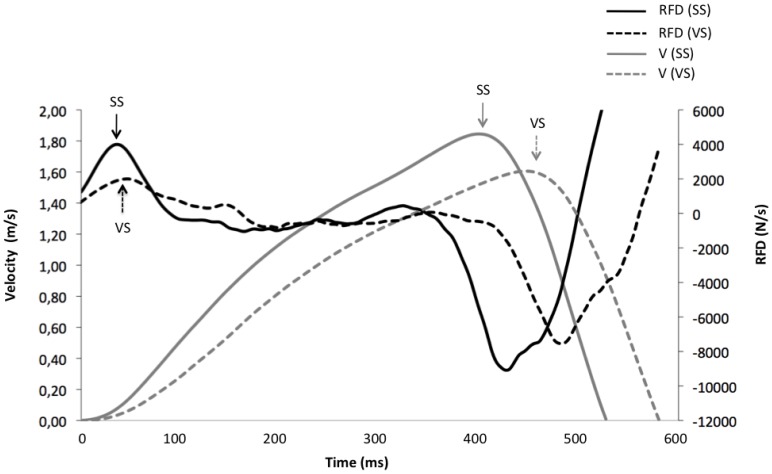
Bench press performance. Examples of the bench press performance in one representative subject for the VS (dashed line lines) and SS (solid lines) conditions. RFD, rate of force development; V, velocity. RFD and V are represented in black and grey colors, respectively.

The analysis of the RFDmax showed a significant main effect for condition (F(2,24) = 7.94, P = 0.002; effect size (EZ) = 0.39; observed power (OP) = 92%). Post-Hoc analysis indicated that the RFDmax during SS condition was significantly higher than during the VS and AS conditions (P = 0.017 and P = 0.003, respectively). No significant difference was found between VS and AS conditions. The analysis of the Vmax showed similar results to the RFDmax (Fig. 2). The ANOVA showed a main effect of condition (F(2,24) = 15.50, P = 0.001; EZ = 0.61; OP = 91%), with significantly higher values of Vmax during the SS condition in comparison with the VS and AS conditions (P = 0.002 and P = 0.001, respectively). No significant differences were found between VS and AS conditions. Figure 3 shows an example of a bench press performance in one representative subject for conditions VS and SS.

The ANOVA of onset movement showed a significant main effect for condition (F = 55.12, P<0.0001; EZ = 0.84; OP = 100%). The onset movement decreased in the IS+AS condition in comparison with the VS condition (P<0.0001) and this reduction was more pronounced in the presence of the startle stimulus (SS vs AS P<0.0001; SS vs VS P<0.0001). The analysis of the movement duration revealed a significant main effect for condition (F (2,24) = 13.2, P = 0.004; EZ = 0.56; OP = 99%). The movement duration was significantly shorter for the SS than for the VS and AS conditions (P<0.0001 and P = 0.003, respectively).

The onset movement did not correlate with the Vmax or RFDmax.

The analysis of the barbell displacement did not reveal any significant differences across conditions.

### Startle effects on EMG characteristics

**Figure 4:**
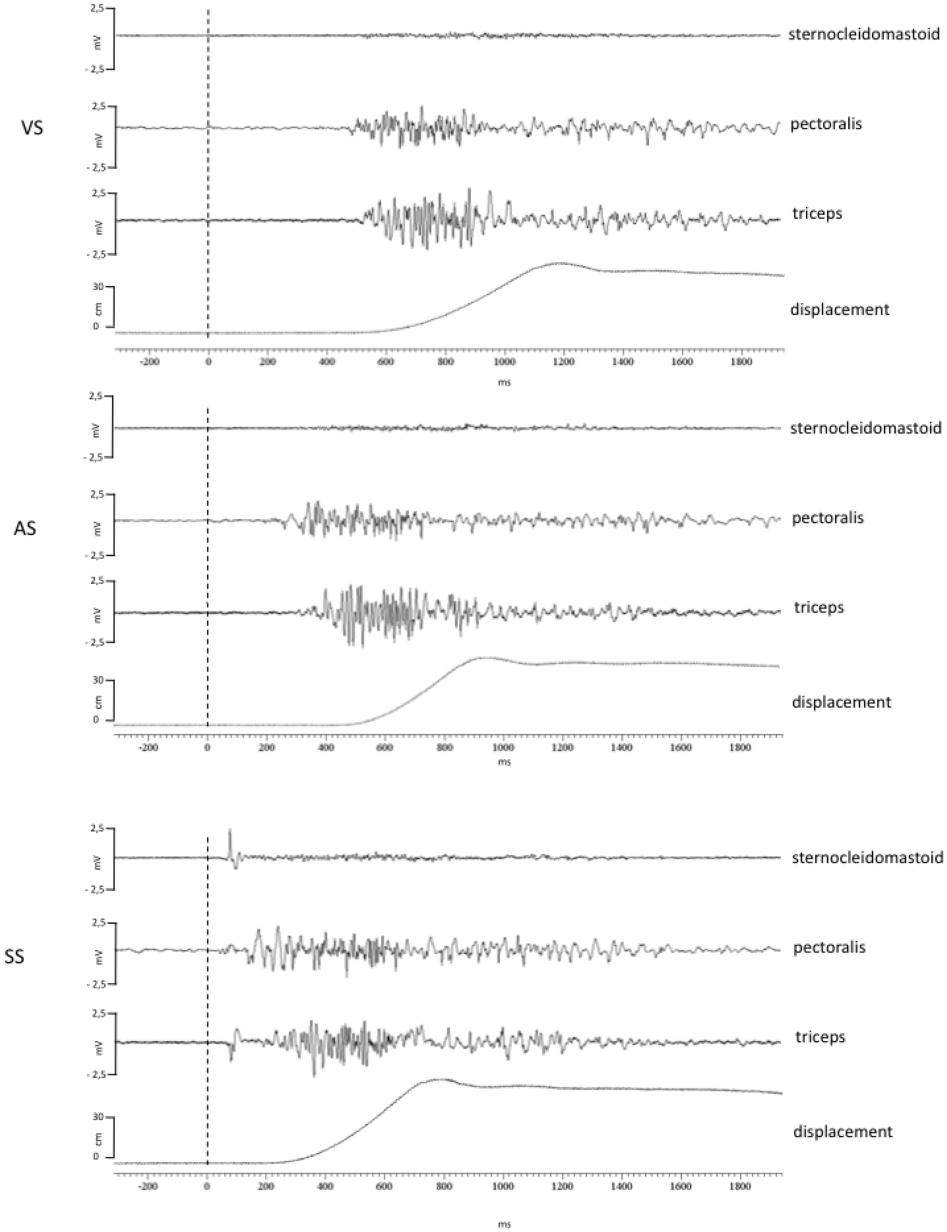
EMG recordings. EMG activity and barbell displacement during the bench press in a subject. (VS) imperative visual stimulus; (AS) visual plus auditory stimulus; (SS) visual plus startle auditory stimulus. Note the sternocleidomastoid burst during the SS condition.

The EMG recordings indicated a homogeneous pattern of muscle activation during the bench press across the subjects. This pattern was characterized by an initial activation of pectoralis, followed by triceps activation. This pattern of activation remained unaffected across the three experimental conditions. For the SS condition this pattern was accompanied by a burst of the SCM in 70% of the trials (see Fig 4).

**Figure 5:**
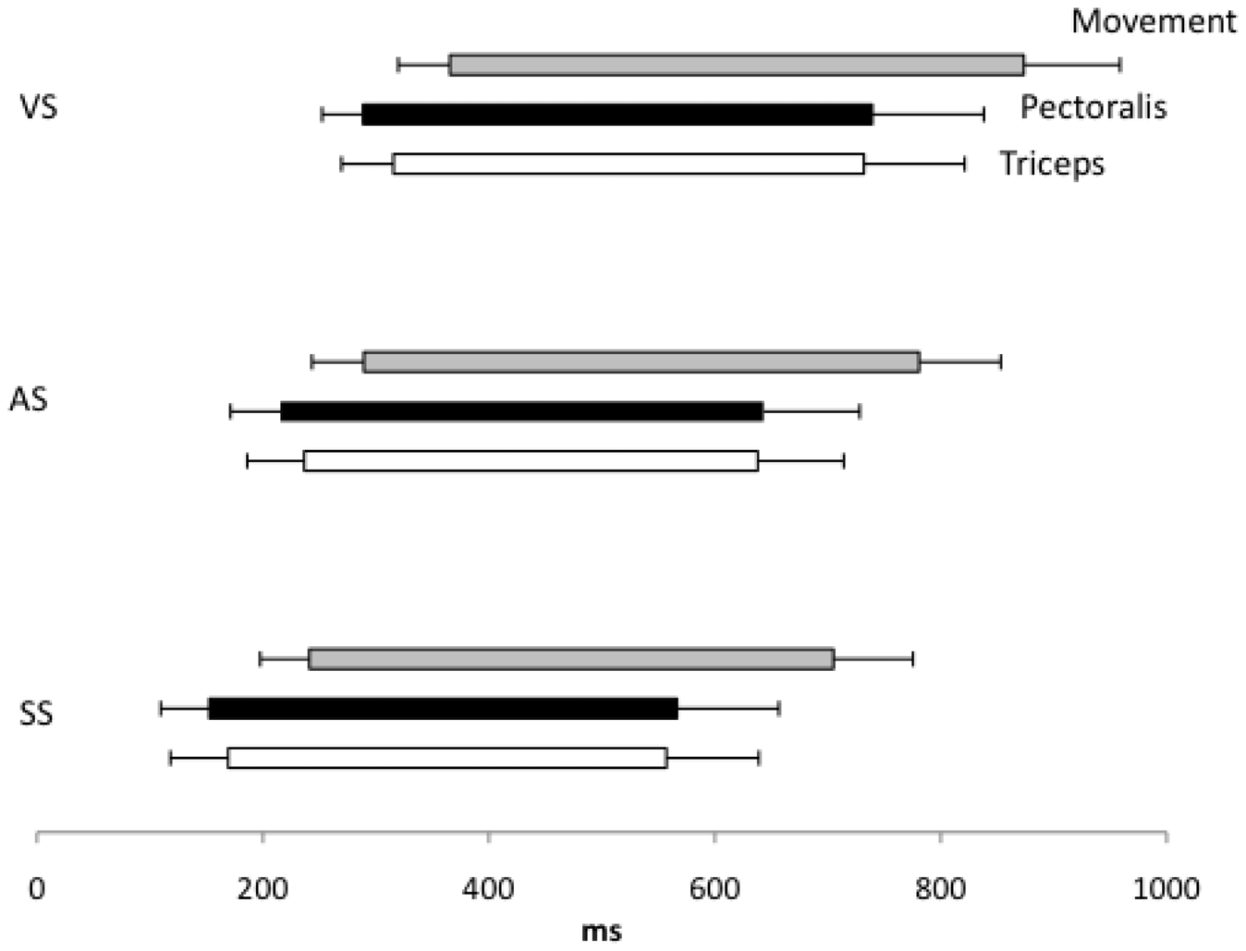
Schematic representation of the mean EMG pattern and movement displacement during concentric bench press across conditions. (VS) imperative visual stimulus; (AS) visual plus auditory stimulus; (SS) visual plus startle auditory stimulus; the leftward extent of the bars represents the mean onset latency, the horizontal line shows +1 S.D. The length of the bars represents the duration of the EMG bursts and the movement duration, and the horizontal lines at the right side of the bar show +s.D.

The time between the activation of the pectoralis and triceps did not change significantly between the conditions in contrast with the activation onset latency of the pectoralis (F(2,24) = 54.82 P<0.0001; EZ = 0.86; OP = 100%) and triceps (F = 83.074 P<0.0001; EZ = 0.89; OP = 100%) (Fig.5). The onset latencies were slower in the VS compared with the AS condition (P = 0.001 and P<0.0001 for pectoralis and triceps, respectively) and in the VS compared with SS condition (P<0.0001 for both muscles). In addition, the onset latencies for the SS condition were significantly faster than for the AS condition (P<0.0001 for both muscles). The analysis of the duration of the muscle activation showed a main effect of condition for pectoralis and triceps (F(2,24) = 6.13, P = 0.031; EZ = 0.41; OP = 82% and F(2,24) = 3.61, P = 0.046; EZ = 0.26; OP = 60%), respectively). During the SS the duration of the EMG activations were significantly shorter for both muscles in comparison with the VS (P = 0.03 for the pectoralis and P = 0.041 for the triceps) and the AS (P = 0.03 and P = 0.44 for the pectoralis and triceps respectively).

The onset EMG latency activation did not correlate with the Vmax or RFDmax.

Friedman test showed a significant difference of the MD between conditions (P<0.0001) and Wilcoxon tests indicated that the MD during the SS condition was significantly longer than the VS and AS conditions (P = 0.003 for both comparisons).

The analysis of the RMS did not reveal any significant differences across conditions for both muscles.

### Baseline blocks

RFDmax comparisons for VOL and VS conditions, before and after the seven sets, showed a main effect of block (F(1, 12) = 6.31, P = 0.027; EZ = 0.34; OP = 64%), condition (F(1,12) = 5.63 P = 0.035; EZ = 0.32; OP = 58%), and block*condition interaction (F (1,12) = 6.97 P = 0.022; EZ = 0.37; OP = 68%). Pos-hoc analysis showed that in Baseline 1 the RFDmax was significantly higher in the VS condition than in the VOL condition (P = 0.005). This difference was absent in the Baseline 2 due to a significant decrease of the RFDmax in the VS condition (P = 0.002). The analysis of the Vmax for the baseline blocks showed a main effect of block (F(1, 12) = 10.46, P = 0.007; EZ = 0.46; OP = 84%) but not a significant effect of condition nor block*condition interaction. The Vmax, grouping all conditions, was significantly lower for baseline 2 in comparison with baseline 1.

## Discussion

The main propose of this study was to explore the effects of auditory startling and non-startling stimuli on rate force development and reaction time during the performance of an explosive dynamic contraction. Our findings show an increase in the RFD and a decrease in reaction time when a startling auditory stimulus was administered together with a visual imperative stimulus, during concentric bench presses. In contrast, this improvement was absent with a non-startling stimulus. These observations point out to specific effects of loud auditory stimulation on the rate of force development.

### RFDmax and Vmax

During the concentric bench press exercise RFDmax and Vmax were higher in response to a startling auditory stimulus compared with a non-startling one. This indicates that the subjects were unable to maximally recruit the motor unit pool during a fast dynamic contraction in absence of the startling stimulus. The higher RFDmax and Vmax evoked by the starling stimulus could reflect an additional increment in the recruitment of the motor units and/or their discharge rates. The latter factor seems to be the more plausible since a recent study suggested that the velocities during fast dynamic or ballistic isometric contractions are associated with the motor units discharge rates [13]. Our results expand previous findings that show an startle dependent increase in the RFD during a maximal ballistic isometric grasp action [8], indicating that this enhancement is not constrained to small muscle groups nor to isometric contractions.

A possible mechanism underlying the RFDmax and Vmax improvements is the startle reaction, a reflex reaction generated in the brainstem in response to unexpected stimuli of various modalities, and most commonly to loud acoustic stimuli [14], [15]. In our study, the elicitation of the startle reaction was also observed in the SCM muscle, the best muscle for detection of a startle-related EMG activity [16], [17]. The increases observed in the RFDmax and Vmax may be mediated by the same mechanisms underlying the startle reaction. Previous studies have indicated that the startle reaction involves the giant neurons in the caudal pontine reticular nucleus that connect to interneurons and motor neurons in the brainstem and spinal cord via the reticulospinal tract [18]. Thus it is likely that the loud stimulus activated the reticulospinal tract, leading to an increase of the discharge rates of motor neurons and resulting in enhanced RFDmax and Vmax.

In addition to the startle reaction, the auditory nature of the stimulus could also enhance the motorneurons excitability due to a decrease in presynaptic inhibition, a phenomenum known as audiospinal facilitation [9]. In line with this idea, audiospinal facilitation could also account for the RFDmax and Vmax increases. However, the audiospinal facilitation is also evoked by non-startling auditory stimuli but in our study there were no differences of the RFDmax and Vmax between the visual and the non-startling auditory conditions. Therefore, it is unlikely that audiospinal facilitation plays a role in the startle induced changes of RFDmax and Vmax.

The results of the movement delay (MD) provide an alternative explanation. The MD was longer for the startle condition in comparison with the visual and auditory non-startling conditions. Although electromechanical delay has been shown to increase as a result of muscle fatigue [19] and to be inversely correlated with rate of force development [20], it is unlikely that both factors account for the MD increase in the startle condition. Muscle fatigue could affect the MD not only during the startle but during all three conditions. In addition, the startling stimulus induces the highest RFD and longest MD. We suggest that the longer MDs are due to an earlier EMG activation that is evoked by the startle reaction. It is plausible that this earlier EMG activation does not reach the minimum intensity in order to overcome the load that is to be lifted, and thus, increases the MD. However, this activation is followed by the voluntary neural drive allowing the subject to initiate the movement. Therefore, the EMG recording during the startle condition could be the summation of the startle reflex and the voluntary response, pre-activating the muscles involved in the task. Two indirect evidences point out to the summation of the reflex and voluntary responses: i) the average duration of the EMG activation of pectoralis during the bench-press was of ∼400 ms, longer than the duration of 200 ms that has been reported [21], thus indicating a voluntary muscle activation and ii) when the subjects were questioned about their perception of the movement, a general impression was that the startle stimulus helped them to accelerate the intended movement. In contrast another study showed that subjects were not aware that the startle stimulus makes them move faster compared with non-startle stimuli during a wrist flexion movement without external load [11]. In the same study the subjects stated that in presence of a startle stimulus, something other than their own will was making them move. It is likely that the startle reaction was enough to initiate and execute the wrist movement in contrast with our study in which a voluntary and “aware” contraction was necessary to perform the bench press movement.

An important question to be addressed is whether the startle stimulus leads to a higher dynamic performance in comparison with a self-initiated movement (as in the VOL condition). In our study, a direct comparison between the SS and VOL conditions could be misleading for the following reasons: i) the values of the SS condition correspond to an average of approximately 24 repetitions vs. 5 repetitions for the VOL condition, and ii) the significant lower values obtained in the second baseline block in comparison with the first one indicated that the 7 sets lead to some form of fatigue. Nevertheless, indirect evidence indicates that the startle condition lead to the highest performance in comparison with the self-initiated movement. The RFD values during the first baseline block were higher for the VS compared with the VOL condition. In addition, RFDmax and Vmax values were higher for the SS compared with the VS condition. These results suggest that a startle auditory stimulus induces performance improvements beyond those that can be achieved by voluntary effort alone.

It should be noted that both Vmax and RFDmax are parameters with high functional significance in fast muscle contractions. The Vmax is considered one of the most important performance criteria of a bench press movement [22], while the RFDmax allows the subject to reach a higher level of muscle force in the early phase of muscle contraction [4]. Although more studies are needed, the startle stimulus could be used as a new paradigm to explore neural adaptations in sports that require explosive movements in addition to different resistance training programs.

### Movement duration

During the startle condition the movement duration was reduced without observed changes in the barbell displacement. Our results are in contrast with a previous study in which subjects were asked to respond to an auditory go signal with either a 20°, 40°, or 60° elbow extension to a fixed target from a fixed starting position of 90° of flexion at the elbow [23]. In this study, the startle stimulus did not increase the peak velocity or movement duration for the arm extension action. However, the instructions used emphasized speed and accuracy of movement, which could compromise high speed in benefit of accuracy.

Our findings showing that the startle condition induced the shortest movement duration and the highest Vmax and RFDmax could be due to a stronger activation of the muscles involved in the movement. However, the RMS of the EMG recordings did not reveal any changes across the three conditions, although it is possible that the high variability of the RMS values between subjects could account for the lack of statistical significant differences. The source of this variability may result from the different absolute resistance loads, used to achieve maximal power, across the subjects. Thus, we cannot rule out an increase in the muscle activation as a result of the startle reaction. In addition, the duration of the pectoralis activation was shorter during this condition compared with the visual and non-startling auditory conditions.

### Reaction time and EMG pattern

When a non-startling auditory stimulus was presented simultaneously with the visual imperative stimulus, the onset of movement and EMG and activation was reduced. This effect could be attributed to intersensory facilitation, a phenomenon in which the reaction time to a stimulus in one sensory modality is shortened significantly if the reaction stimulus is paired with a stimulus in another modality (an accessory stimulus) that is presented in close temporal proximity [24].

In addition, our study showed that the onset of EMG activation and movement onset in response to a startling stimulus is faster compared to a non-startling stimulus. The onset of EMG activation for both pectoralis and triceps muscles in trials containing SS occurred approximately 140 ms earlier than in trials containing only the VS and 60 ms earlier for non-startling auditory stimuli. A “stimulus-intensity effect”, i.e. a decrease in reaction time with an increase in the intensity of a stimulus [25], may explain the decrease in reaction time between the two auditory conditions. However, the magnitude of the reaction time is larger than that expected for the increase in stimulus intensity. This is in line with the observations of Carlsen and colleagues [17], who reported that startle produces early response latencies that are distinct from stimulus intensity effects.

The EMG analysis showed a constant pattern of activation across conditions. Only one subject felt that in presence of the startle stimulus the control of the movement was disturbed. However, in this case as in the rest of the subjects, there was no sign that the startle had disrupted the pattern of the voluntary responses during the startle condition. The short reaction time and unaffected electromyography pattern supports the hypothesis that the startle effect is due to triggering of subcortical motor programmes [11]. Nonetheless, the findings of relatively long activation onsets of the pectoralis (152 ms) and triceps (169 ms) in comparison with the SCM muscle (71 ms) during the startle condition do not support this hypothesis, and the long premotor reaction time speaks against a summation of startle reflex and voluntary drive. However, we should point out that the execution of a bench-press movement may involve associated postural adjustments (APA), due to the lying position and the control of the bar over the chest, that could delay the onset of the muscles that act as a primer movers. It has been reported that these time differences vary from 50 to more than 150 ms according to the task [26], [27]. Therefore, the EMG onset values in our study could be compatible with the hypothesis of a motor program triggered at a subcortical level. Another alternative explanation is that the startle reaction provides sufficient ascending activation via reticulo-thalamo-cortical pathways to involuntarily trigger the prepared response at a cortical level [28].

It is important to note that the onset of EMG activation and the onset of movement were not correlated with Vmax or RFDmax for any experimental condition. This is line with previous studies that did not find a correlation between reaction time and force response [29], [30] indicating that temporal and dynamic aspects of a motor response are independent of each other [31]. Thus, reaction time changes are not necessarily associated with changes in RFD and startle induced facilitation of movement onset, while EMG activation does not account for the increase in the movement dynamics, suggesting that both parameters could reflect different processes involved in a motor response.

### Perspectives

In summary, our study shows that a startle auditory stimulus can lead to an increase in the peak of velocity, peak of rate force development and a faster reaction time during an explosive dynamic contraction. Our results suggest that loud auditory stimulation enhances fast dynamic muscle contractions. A startle stimulus could be used as a new paradigm to explore neural adaptations to resistance training.
